# Real-World Treatment Patterns and Outcomes Amongst Patients with Resectable Gastric and Gastroesophageal Junction Cancer in the United States

**DOI:** 10.3390/cancers17213546

**Published:** 2025-11-01

**Authors:** Vishal Patel, Michael Baglio, Di He, Niamh Hogan, Lauren Damato, Heide Stirnadel-Farrant

**Affiliations:** 1 Oncology Outcomes Research, AstraZeneca, Cambridge CB2 0AA, UK; heide.stirnadel-farrant@astrazeneca.com; 2 US Medical Affairs, AstraZeneca, Gaithersburg, MD 20878, USA; 3 Global Oncology Data & Analytics, AstraZeneca, Gaithersburg, MD 20878, USA; 4 Global Medical Affairs, AstraZeneca, Cambridge CB2 0AA, UK; 5 Flatiron Health, New York, NY 10013, USA

**Keywords:** gastric cancer, gastroesophageal junction cancer, immunotherapy, chemotherapy

## Abstract

**Simple Summary:**

This study looked at treatments given before and after surgery and outcomes for patients in the US with resectable gastric or gastroesophageal cancer (GC/GEJC) in routine clinical practice (real-world) rather than a clinical trial. Data from electronic health records were studied for adult patients diagnosed with resectable GC/GEJC between 1 January 2016 and 1 January 2023. This real-world study found the most common treatments used were those recommended in clinical guidelines at the time of the study. However, the use of treatments before and after surgery for patients with resectable GC/GEJC was lower than expected. The length of time before an event where the patient’s disease worsens significantly or the patient dies, and the length of time patients were alive after treatment, were both poor, underscoring the need for new treatment options to improve outcomes.

**Abstract:**

Background: Resectable gastric and gastroesophageal junction cancer (GC/GEJC) treatment patterns in the real-world are poorly described. This study described real-world perioperative treatment and outcomes for patients in the US with resectable GC/GEJC. Methods: Data from the Flatiron Health Enhanced Datamart were analyzed for adult patients diagnosed with resectable GC/GEJC between 1 January 2016 and 1 January 2023. The primary objective was to describe perioperative treatments (neoadjuvant only, adjuvant only, both). Secondary objectives included real-world event-free survival (rwEFS) and real-world overall survival (rwOS). Results: Data from 1717 patients (901/816 with GC/GEJC) were included. Median age of patients with GC/GEJC was 68.0/69.0 years, 62.4%/83.3% were male, and 97.3%/96.7% had adenocarcinoma, respectively. For GC/GEJC, 71.1%/47.9% underwent surgery, of which 15.6%/70.1% received neoadjuvant treatment only, 26.4%/5.6% received adjuvant treatment only, 25.0%/17.4% received both, and 33.1%/6.9% received no perioperative treatment, respectively. For GC, the most frequent neoadjuvant treatment was FLOT (43.0% neoadjuvant only; 53.8% both) and the most frequent adjuvant treatments were chemoradiotherapy (39.6% adjuvant only) and FLOT (43.1% both). For GEJC, chemoradiotherapy was the most frequent neoadjuvant (66.4% neoadjuvant only; 67.6% both) and adjuvant only (54.5%) treatment. When patients received both, the most frequent adjuvant treatment was nivolumab (45.6%). For GC/GEJC, median rwEFS (95% CI) was 29.1 (24.7–38.7)/20.8 (17.4–23.7) months for patients who had planned or cancelled surgery and 11.3 (9.6–13.5)/12.7 (11.6–15.4) months for patients without planned surgery. Median rwOS (95% CI) was 50.9 (43.7–62.4)/38.6 (31.4–47.2) months for patients who had planned or cancelled surgery and 15.4 (13.1–18.6)/21.0 (17.6–22.6) months for patients without planned surgery. Conclusions: Real-world data showed lower use of perioperative treatments for resectable GC/GEJC than expected. rwEFS and rwOS remain poor. Optimization of perioperative treatments is needed to improve long-term outcomes.

## 1. Introduction

Globally, stomach cancers—including gastric cancer and gastroesophageal junction cancer (GC/GEJC)—have the fifth highest incidence of any cancer type by site and are the fifth most common cause of cancer-related deaths [1]. Incidence of stomach cancers varies substantially by geographic region, with the highest prevalence observed in Eastern Asia and Eastern Europe [1,2,3]. Within the United States (US), GC/GEJC accounted for an estimated 1.3% (26,890) of newly diagnosed cancer cases and was predicted to result in 1.8% (10,880) of cancer deaths in 2024 [4,5]. Despite recent advances in surgical and therapeutic treatment options, overall 5-year relative survival for GC/GEJC remains poor, at less than 20% worldwide and ~36% in the US, largely due to limited early detection and screening resulting in patients with GC/GEJC often being diagnosed in the locally advanced or metastatic stage [4,5,6,7]. By disease stage, the 5-year relative survival in the US is 75.4%, 35.8%, and 7.0% for localized, regional, and distant disease, respectively [5]. In addition, many patients experience recurrence after curative surgery [8,9,10,11].

Treatment guidelines for medically fit patients with resectable GC/GEJC recommend endoscopic or surgical resection, which can be combined with pre/post-operative chemoradiation and/or perioperative (neoadjuvant and adjuvant) treatments [10,12,13,14,15]. The National Comprehensive Cancer Network^®^ (NCCN^®^) Clinical Practice Guidelines in Oncology (NCCN Guidelines^®^) for the treatment of resectable GC (version 2.2025) recommend surgery plus perioperative chemotherapy with FLOT (5-fluorouracil, leucovorin, oxaliplatin, and docetaxel) or fluoropyrimidine and oxaliplatin, with postoperative chemotherapy plus chemoradiotherapy recommended for patients who received less than a D2 lymph node dissection [12]. In addition, immune checkpoint inhibitor regimens, including neoadjuvant durvalumab (anti-programmed cell death ligand-1 antibody) and neoadjuvant tremelimumab (anti-cytotoxic T-lymphocyte-associated antigen 4 [CTLA-4] antibody), nivolumab (anti-programmed cell death-1 [PD-1] antibody) and ipilimumab (anti-CTLA-4 antibody) followed by nivolumab, and pembrolizumab (anti-PD-1 antibody), are now recommended for neoadjuvant or perioperative use in patients with microsatellite instability-high/mismatch repair deficient (MSI-H/dMMR) tumors. The NCCN Guidelines^®^ (version 3.2025) for the treatment of GEJC recommend perioperative chemotherapy with FLOT as the preferred treatment option [13]. Recent guideline updates can be attributed to new readouts, such as the ESOPEC study in 2024, which showed perioperative FLOT improved overall survival (OS) versus neoadjuvant 41.4 Gy plus carboplatin/paclitaxel (CROSS, a chemoradiotherapy) [16], and neoadjuvant chemoradiotherapy is now considered an alternative recommendation. Similarly to their use in GC, neoadjuvant or perioperative immune checkpoint inhibitors should also be considered for use in patients with GEJC with MSI-H/dMMR tumors.

Despite the evolving treatment landscape of resectable GC/GEJC, current data on real-world treatment patterns and outcomes in patients with resectable GC/GEJC are limited, largely because real-world studies often focus on treatment use and outcomes in patients with advanced stage disease. Amongst the limited real-world data for resectable GC are the results from a study of patients in the US with GC between 2004 and 2016, which assessed GC treatment trends over time and any correlation between outcomes and patients receiving guideline concordant care, grouped by disease stage [17]. The study concluded that patients receiving care in accordance with treatment guidelines had significantly improved 3-year OS compared with those who did not, regardless of disease stage [17]. More recently, the GARIBALDI study described perioperative treatment patterns in patients with Stage II–IVA GC/GEJC in the US, where the data indicated an unmet need for additional perioperative treatment options in GC/GEJC [18]. Given this lack of real-world data regarding the treatment of patients with resectable GC/GEJC, the aim of this retrospective, observational study was to describe real-world perioperative treatment patterns and outcomes for patients with resectable GC and GEJC within the US.

## 2. Materials and Methods

### 2.1. Data Source

This real-world, retrospective study used data from the Flatiron Health Enhanced Datamart (Flatiron Health^®^, New York, NY, USA) for advanced gastric, esophageal, and gastroesophageal cancer, which features comprehensive early gastric, esophageal, and gastroesophageal cancer patient-level data. The Flatiron dataset contains de-identified patient-level data derived from electronic health records from over 280 cancer clinics across the US.

### 2.2. Study Design and Population

**Figure 1:**
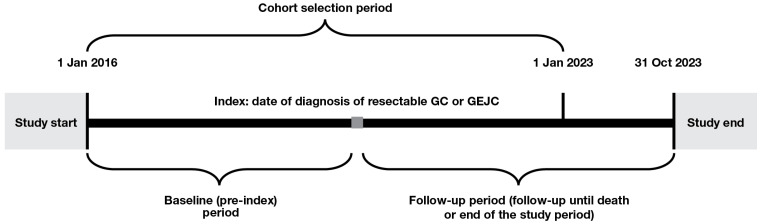
Study design. GC, gastric cancer; GEJC, gastroesophageal junction cancer.

The study included adult patients (≥18 years of age) diagnosed with resectable GC or GEJC between 1 January 2016 and 1 January 2023 (the date of diagnosis is referred to, hereafter, as ‘index’). Patients were required to have cancer staging recorded at diagnosis with Tumor, Node, and Metastasis stage of either ≥T2, N0–3, and M0 or T0–4, N1–3, and M0. Patients were excluded if they had any other cancer, metastatic disease or gastrointestinal stromal tumors prior to index; had been a clinical study participant before; or had ever been diagnosed with lymphoma. The study design is shown in Figure 1.

### 2.3. Study Objectives and Assessments

The primary objective was to describe the treatment regimens received by patients diagnosed with resectable GC/GEJC in the perioperative setting. Secondary objectives included describing baseline demographics and characteristics across treatment groups and patient outcomes, such as real-world event-free survival (rwEFS; defined as the time from diagnosis to either the date of surgery cancellation due to disease progression, first locoregional recurrence, metastatic disease, or death, whichever occurred earliest) and real world OS (rwOS; defined as the time from diagnosis to death). Subgroup analyses were performed, where a sufficient sample size was available, for rwEFS and rwOS stratified by Eastern Cooperative Oncology Group performance status (ECOG PS) and tumor stage as these are known prognostic factors for GC or GEJC. Patient demographics and clinical characteristics were assessed at index and included age, sex, race and ethnicity, body mass index, practice type (community vs. academic setting, as defined by Flatiron Health), histology, disease stage (based on T stage), baseline ECOG PS, and laboratory tests for carcinoembryonic antigen and cancer antigen 19-9. Perioperative treatments were described amongst patients who received neoadjuvant treatment only, adjuvant treatment only, and patients who received both neoadjuvant and adjuvant treatment. A subgroup analysis of patients diagnosed post-2018 was undertaken to assess the impact of FLOT on the treatment landscape subsequent to the FLOT4 study, which established the superior benefit of perioperative treatment with FLOT compared with the previous standard of care [19]. Treatments included FLOT, FOLFOX, chemoradiotherapy, doublet chemotherapy, chemotherapy plus HER2-targeted therapy, other chemotherapy, or radiotherapy. When there were five or fewer patients for any study variable, data were not reported to maintain confidentiality.

### 2.4. Statistical Analysis

Descriptive statistics were used within this non-comparative study. Time from index to event outcomes were estimated using Kaplan–Meier methods, with medians and related 95% confidence intervals (CIs) presented.

## 3. Results

### 3.1. Patient Demographics and Clinical Characteristics

A total of 1717 patients (901 with GC and 816 with GEJC) were included in the study (Table 1 and Table S1). Demographics and clinical characteristics of patients included in the analysis are presented in Table 1. The median age was 68.0 (interquartile range [IQR]: 59.0–76.0) years and 69.0 (IQR: 61.0–75.0) years for patients with GC and GEJC, respectively. A higher proportion of patients were male (GC: 562 [62.4%]; GEJC: 680 [83.3%]). Adenocarcinoma was the most frequently observed histology (GC: 877 [97.3%]; GEJC: 789 [96.7%]). For patients with GC, 299 (33.2%) and 202 (22.4%) had an ECOG PS of 0 or 1, respectively (ECOG PS unknown: 331 [36.7%] patients). For patients with GEJC, 287 (35.2%) and 220 (27.0%) had an ECOG PS of 0 or 1, respectively (ECOG PS unknown: 252 [30.9%] patients).

At index, 785 (87.1%) patients with GC and 652 (79.9%) patients with GEJC were from the community setting, and 116 (12.9%) and 164 (20.1%), respectively, were from the academic setting.

### 3.2. Surgery and Perioperative Treatments

Among patients with GC or GEJC, 641 (71.1%) and 391 (47.9%) underwent surgical resection, respectively (Table 1); surgery was planned for an additional 42 (7.9%) and 63 (10.6%) patients, respectively, but was cancelled due to disease progression. Subtotal (partial) gastrectomy (419 [65.4%]) and esophagogastrectomy (268 [68.5%]) were the most frequent types of resections received for GC and GEJC, respectively.

Of the patients who underwent surgical resection, for GC and GEJC, respectively, 100 (15.6%) and 274 (70.1%) received neoadjuvant treatment only, 169 (26.4%) and 22 (5.6%) received adjuvant treatment only, 160 (25.0%) and 68 (17.4%) received both, and 212 (33.1%) and 27 (6.9%) received no perioperative treatment.

Baseline demographics and clinical characteristics for patients with GC or GEJC by treatment setting are shown in Tables S2 and S3. For patients with GC, the most frequent neoadjuvant treatment was FLOT, which was administered to 43/100 (43.0%) patients receiving neoadjuvant treatment only, and 86/160 (53.8%) patients receiving both neoadjuvant and adjuvant treatments (Table 2). Of the 129 patients treated with FLOT, 118 were diagnosed with GC after 2018. As an adjuvant treatment, FLOT was administered to 69/160 (43.1%) patients with GC receiving both neoadjuvant and adjuvant treatment, with 60/69 diagnosed after 2018, and to <3% of the 169 patients who were treated with adjuvant therapy alone (Table 2). For patients with GEJC, the most frequent treatment was chemoradiotherapy amongst both patients receiving neoadjuvant treatment only (182/274 [66.4%]) and those receiving adjuvant treatment only (12/22 [54.5%]). When patients received both neoadjuvant and adjuvant treatment, chemoradiotherapy remained the most frequent neoadjuvant treatment (46/68 [67.6%]), and the most frequent adjuvant treatment was nivolumab (31/68 [45.6%]; Table 3).

Neoadjuvant and adjuvant treatment patterns for patients diagnosed post-2018 up to 1 January 2023, are provided in Supplementary Table S4 and Table S5, respectively. Of note, amongst patients with GC diagnosed post-2018 who underwent surgery, 28.9% received neoadjuvant FLOT, while 14.7% received adjuvant FLOT. Amongst patients with GEJC, 8.5% received neoadjuvant FLOT, while 2.7% received adjuvant FLOT.

### 3.3. Patient Outcomes

**Figure 2:**
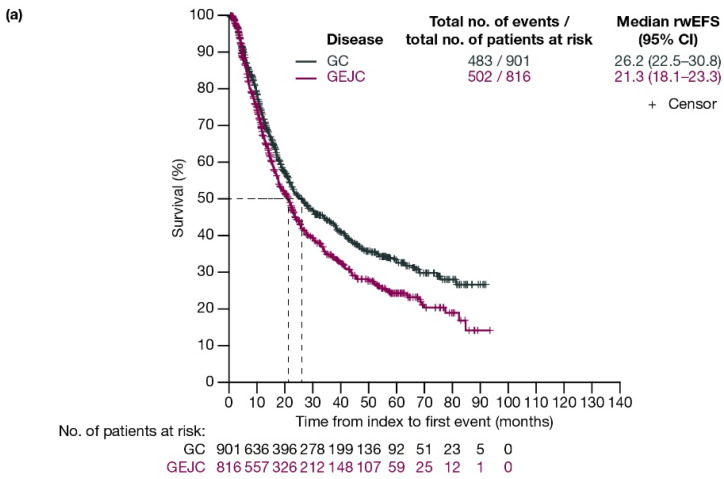

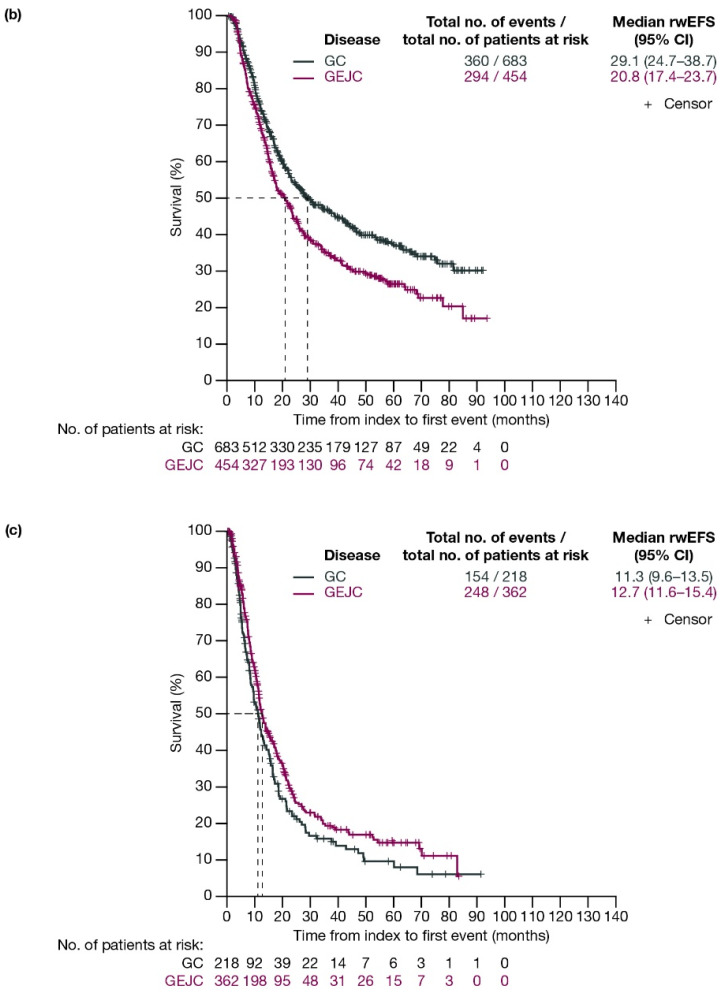
Kaplan–Meier curves of rwEFS for patients with GC or GEJC (**a**) in the full analysis population, (**b**) who had surgery planned (including those who had surgery cancelled due to progression), and (**c**) who did not have surgery planned. Dashed lines represent the medians. CI, confidence interval; GC, gastric cancer; GEJC, gastroesophageal junction cancer; rwEFS, real-world event-free survival.

For patients with GC and GEJC, respectively, median rwEFS was 26.2 (95% CI: 22.5–30.8) months and 21.3 (95% CI: 18.1–23.3) months (Figure 2a). When stratified by planned surgery, median rwEFS (95% CI) for patients with GC and GEJC, respectively, was 29.1 (24.7–38.7) months and 20.8 (17.4–23.7) months in those who had surgery planned (including those who had surgery cancelled due to progression; Figure 2b), and 11.3 (9.6–13.5) months and 12.7 (11.6–15.4) months in those who did not have surgery planned (Figure 2c).

When stratified by ECOG PS, median rwEFS (95% CI) for patients who underwent surgery or had surgery cancelled was 40.0 (27.1–67.0) months for GC and 21.0 (15.6–26.4) months for GEJC with ECOG PS 0; 24.7 (17.0–36.4) months for GC and 15.6 (11.9–23.7) months for GEJC with ECOG PS 1; and 19.6 (8.5–not evaluable [NE]) months for GC and 17.1 (14.3–41.5) months for GEJC with ECOG PS ≥2 (Figure S1).

When stratified by disease stage (T2, T3, T4), median rwEFS (95% CI) for patients with GC and GEJC who underwent surgery or had surgery cancelled was 58.0 (42.1–NE) months for GC and 36.8 (17.8–NE) months for GEJC for T2; 27.8 (22.1–46.4) months for GC and 18.0 (15.8–22.6) months for GEJC for T3; and 18.4 (14.2–24.6) months for GC and 17.8 (14.8–NE) months for GEJC for T4 (Figure S2).

**Figure 3:**
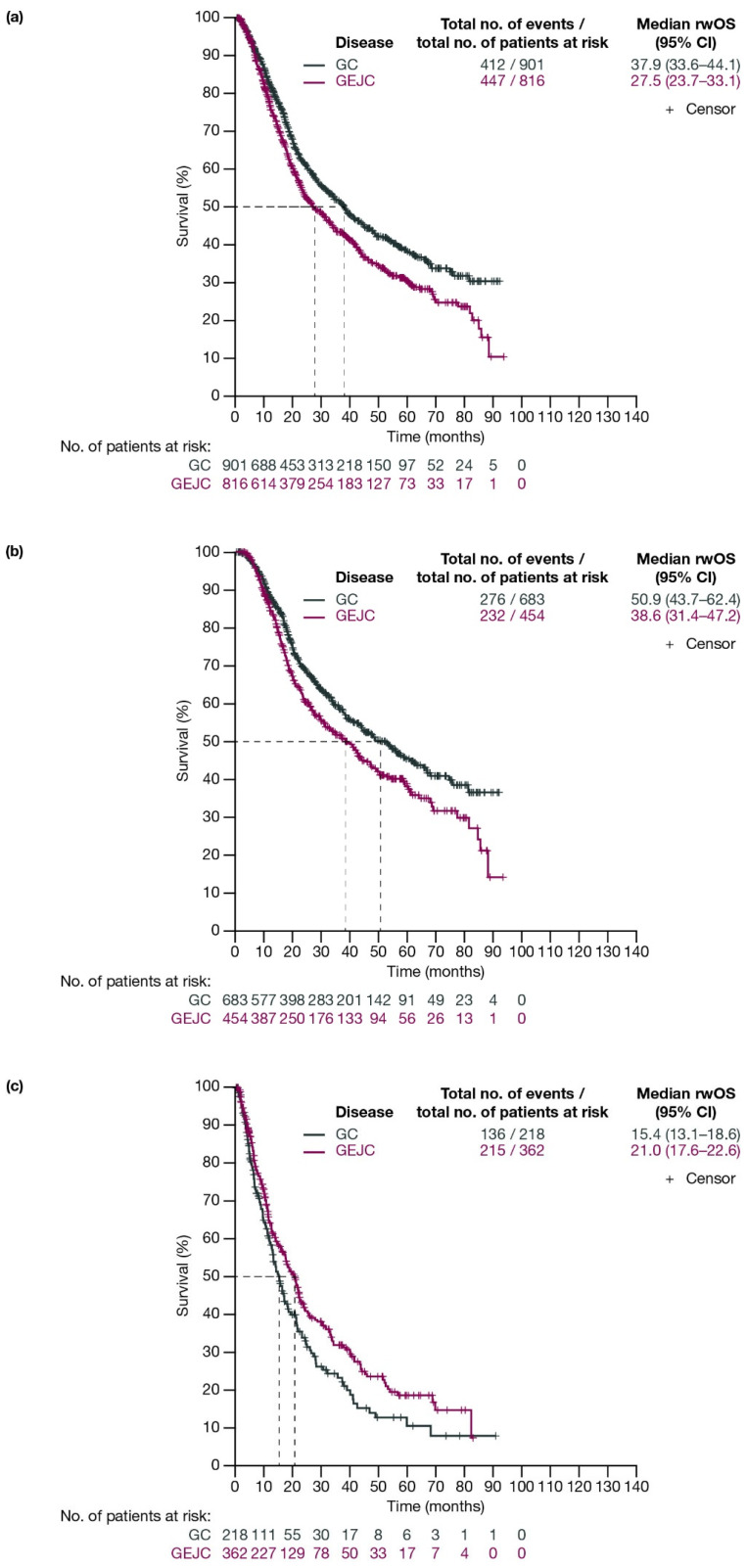
Kaplan–Meier curves of OS for patients with GC or GEJC (**a**) in the full analysis population, (**b**) who had surgery planned (including those who had surgery cancelled due to progression), and (**c**) who did not have surgery planned. Dashed lines represent the medians. CI, confidence interval; GC, gastric cancer; GEJC, gastroesophageal junction cancer; OS, overall survival.

For patients with GC and GEJC, respectively, median rwOS (95% CI) was 37.9 (33.6–44.1) months and 27.5 (23.7–33.1) months (Figure 3a). When stratified by planned surgery, median (95% CI) rwOS for patients with GC and GEJC, respectively, was 50.9 (43.7–62.4) months and 38.6 (31.4–47.2) months among patients who had surgery planned (including those who had surgery cancelled due to progression; Figure 3b), and 15.4 (13.1–18.6) months and 21.0 (17.6–22.6) months in those who did not have surgery planned (Figure 3c).

When stratified by ECOG PS, median rwOS (95% CI) for patients with GC and GEJC who underwent surgery or had surgery cancelled was 61.1 (48.1–NE) months for GC and 40.4 (24.2–49.4) months for GEJC with ECOG PS 0; 34.9 (27.5–67.2) months for GC and 27.6 (19.9–50.6) months for GEJC with ECOG PS 1; and 30.9 (19.7–NE) months for GC and 18.7 (15.8–NE) months for GEJC with ECOG PS ≥ 2 (Figure S3).

When stratified by disease stage (T2, T3, T4), median rwOS (95% CI) for patients with GC and GEJC who underwent surgery or had surgery cancelled was 74.8 (52.6–NE) months for GC and NE (43.5–NE) for GEJC for T2; 62.4 (44.5–NE) months for GC and 31.7 (26.1–41.4) months for GEJC for T3; and 28.9 (20.0–37.9) months for GC and 20.8 (18.0–NE) months for GEJC for T4 (Figure S4).

## 4. Discussion

This retrospective, real-world, observational study evaluated demographics, clinical characteristics, treatment patterns, and outcomes in US patients with resectable GC and GEJC diagnosed between 1 January 2016 and 1 January 2023 from a heterogeneous patient population. In this study, only 71.1% of patients with GC and 47.9% of patients with GEJC underwent resection, while 7.9% and 10.6%, respectively, had planned surgery that was cancelled due to disease progression. Given that surgical resection is a potentially curative therapy, increasing the number of patients able to receive surgery through earlier diagnosis or optimal therapy prior to surgery may benefit patient outcomes [10].

This study identified that within the real-world treatment setting, only a small proportion of patients with resectable GC (25.0%) or GEJC (17.4%) who underwent surgical resection received both neoadjuvant and adjuvant treatment, and around one-third of patients with GC (33.1%) did not receive any perioperative treatment at all. These findings appear surprising, given that treatment guidelines during the study period recommended the use of perioperative chemotherapy for patients with resectable GC and GEJC [10,20,21]. However, the limited use of perioperative therapy could be due to a number of factors that were not assessed in this study, such as comorbidities, physician decision making, or socioeconomic barriers to care; further research into these limitations is warranted.

One of the first perioperative treatments recommended for the treatment of resectable GC/GEJC was ECF (epirubicin, cisplatin, and infused 5-fluorouracil), following results from the MAGIC study in 2006 that demonstrated improved OS and progression-free survival with perioperative ECF compared with surgery alone [22]. In 2012, the CROSS-regimen study showed improved survival (median OS of 49.4 months vs. 24.0 months) with preoperative chemoradiotherapy (carboplatin, paclitaxel, and radiation) versus surgery alone for treatment of resectable GEJC [23]. In 2017, the FLOT4 study established the superior benefit of perioperative treatment with FLOT compared with ECF or ECX (epirubicin plus capecitabine and cisplatin) for resectable GC/GEJC, with an improved median OS of 50 months with FLOT versus 35 months with ECX/ECF [19]. Recently, in 2024, the ESOPEC study further demonstrated an improved OS benefit with perioperative FLOT versus the preoperative chemoradiotherapy regimen from the CROSS-regimen study for patients with esophageal cancer and GEJC, with a median OS of 66 months versus 37 months, and 5-year OS rates of 50.6% versus 38.7%, respectively [16,24,25]. As a result of these studies, treatment guidelines have been updated to recommend perioperative chemotherapy with FLOT as the preferred treatment for resectable GEJC, now consistent with the recommendations for resectable GC [12,13].

This study identified that in the real-world setting, FLOT was the most frequent regimen for patients with GC, received by 43.0% of patients treated with neoadjuvant therapy alone (therefore these patients did not continue with adjuvant FLOT). In patients with GC treated with both neoadjuvant and adjuvant therapy, 53.8% received FLOT in the neoadjuvant setting and 43.1% received FLOT in the adjuvant setting; therefore, approximately half the population did not receive this preferred regimen from the GC guidelines. Use of FLOT in patients with GC as an adjuvant-only therapy was low (<3%), as would be expected for a treatment recommended for perioperative use. When considering patients with GEJC, FLOT use was very low, with just 4.7% of patients receiving FLOT as neoadjuvant therapy alone, and 13.2% (neoadjuvant) and 10.3% (adjuvant) of patients receiving FLOT amongst those who received both neoadjuvant and adjuvant treatment. In the post-2018 subgroup, FLOT use was higher in patients with GC, with almost a third of patients receiving the regimen in the neoadjuvant setting. However, the data for this study were collected prior to the guideline updates for GEJC based on the aforementioned ESOPEC study, which may result in better treatment outcomes in the future.

Demographic data for patients in this study show some evidence that those with GC receiving neoadjuvant therapy alone or both neoadjuvant and adjuvant therapies were more likely to be aged between 50–69 years, have an ECOG PS score of 0, and a tumor stage of T2 or T3 at diagnosis. These data follow expectations, as patients must be diagnosed at an earlier stage to receive perioperative therapies, highlighting the importance of early diagnosis. Furthermore, NCCN Guidelines recommend FLOT for patients with an excellent ECOG PS, aligning with the observed prevalence of ECOG PS 0 in this study.

Chemoradiotherapy was the most frequent neoadjuvant therapy for patients in this study with GEJC, regardless of whether patients received neoadjuvant therapy only (66.4%), or both neoadjuvant and adjuvant therapy (67.6%). Adjuvant therapy was administered less frequently for patients with GEJC compared with GC. For patients with GEJC that received adjuvant therapy only, chemoradiotherapy was the most frequently administered (54.5%). Although the proportion of patients receiving chemoradiotherapy appears high, it should be noted that this was in the adjuvant-only subgroup; among the entire GC population receiving adjuvant therapy, adjuvant chemoradiotherapy was administered to 24.6% (81/329) of patients, a figure closer to that observed elsewhere, such as 16% in the GARIBALDI study [18]. As noted above, survival data from the ESOPEC study have led to updated guidance, with perioperative FLOT now recommended as the preferred course of treatment for resectable GEJC. However, this recent update in the guidelines occurred subsequent to the timeframe of this study, which could account for the higher use of chemoradiotherapy versus FLOT. Additional studies will be needed to examine the impact of this shift in treatment strategies on real-world outcomes for patients with resectable GEJC.

Results from this real-world study identified that the use of immunotherapy other than nivolumab was very low, which reflects the recommendation of such treatments in the current guidelines to restrict their use to MSI-H/dMMR tumors. Few patients with GC/GEJC were treated with neoadjuvant or adjuvant chemotherapy plus immunotherapy, or with any adjuvant immunotherapy other than nivolumab. Nivolumab was the most frequent adjuvant treatment (45.6%) for patients with GEJC who were also treated with a prior neoadjuvant therapy, which is reflective of current guideline recommendations [13]. Adjuvant nivolumab was approved in 2021 by the US Food and Drug Administration for the treatment of completely resected GEJC with residual pathologic disease following neoadjuvant chemoradiotherapy, following the observation of a statistically significant improvement in disease-free survival in the CheckMate 577 study [26,27]. Subsequently, the CheckMate 577 study OS analysis did not meet statistical significance for adjuvant nivolumab versus placebo [28], which could lead to a further shift towards the use of perioperative FLOT in this population.

Clinical studies in patients with resectable GC/GEJC are investigating the use of perioperative immunotherapy. MATTERHORN is an ongoing Phase 3 study designed to assess the efficacy and safety of neoadjuvant/adjuvant durvalumab plus FLOT followed by durvalumab monotherapy versus neoadjuvant/adjuvant placebo plus FLOT followed by placebo in patients with resectable GC/GEJC [29,30]. The primary analysis showed a significant improvement in 2-year EFS (primary endpoint) with durvalumab plus FLOT versus placebo plus FLOT (67.4% vs. 58.5%; hazard ratio [HR]: 0.71 [95% CI: 0.58–0.86]; *p* < 0.001) [29]. Median OS (95% CI) was not reached (40.7 months–not reached) in the durvalumab plus FLOT arm and was 32.8 months (27.9–not reached) in the placebo plus FLOT arm [29]. The final OS analysis was also more recently presented and showed a statistically significant and clinically meaningful improvement in OS with durvalumab plus FLOT versus placebo plus FLOT (HR: 0.78 [95% CI: 0.63–0.96]; *p* = 0.021); these recent results from MATTERHORN support the use of perioperative durvalumab plus FLOT as a potential first line standard of care in resectable GC/GEJC.

In addition, a third interim analysis conducted on a cohort of patients with locally advanced, resectable GC/GEJC treated with neoadjuvant/adjuvant FLOT with or without pembrolizumab, which was added to the KEYNOTE-585 study after the main study was ongoing, showed that treatment with neoadjuvant/adjuvant pembrolizumab plus FLOT improved pathologic complete response. Median EFS (95% CI) was not reached (28.2–not reached) at data cutoff for the pembrolizumab plus FLOT arm and 30.9 (22.8–not reached) months for the placebo plus FLOT arm; the HR was 0.79, however the 95% CI was 0.52–1.22 [31]. In this real-world study, median rwEFS (95% CI) was 26.2 (22.5–30.8) months for patients with GC, and 21.3 (18.1–23.3) months for patients with GEJC. Although not directly comparable, rwEFS observed in this real-world study was similar to the placebo plus chemotherapy arm of the main KEYNOTE-585 study, which reported a median EFS of 25.3 (95% CI: 20.6–33.9) months for patients with locally advanced GC/GEJC treated with surgery [32]. Notably, in KEYNOTE-585, patients treated with both neoadjuvant and adjuvant pembrolizumab had longer median EFS (44.4 months; 95% CI: 33.0–not reached) compared with placebo (HR: 0.81; 95% CI: 0.67–0.99); however, this difference was not statistically significant [32]. Median OS (95% CI) results from the KEYNOTE-585 study were 58.0 months (41.5–not reached) in the placebo arm and 60.7 months (51.5–not reached) in the pembrolizumab arm (HR: 0.90 [95% CI: 0.73–1.12]; *p* = 0.174) [32].

In this real-world study, median rwEFS (95% CI) for patients who had surgery planned (including those who had surgery cancelled due to progression) was 29.1 (24.7–38.7) months for patients with GC and 20.8 (17.4–23.7) months for patients with GEJC. For those patients who did not have surgery planned, median rwEFS (95% CI) was 11.3 (9.6–13.5) months for GC and 12.7 (11.6–15.4) months for GEJC. There is evidence of a difference in EFS amongst patients with GC with and without planned surgery, but no comparable evidence of a difference amongst patients with GEJC with and without planned surgery. As expected, patients with more advanced tumors or a higher ECOG PS at baseline generally had reduced rwEFS and rwOS. However, as there was no comparative analysis undertaken, any causality cannot be determined currently. Median rwOS remained poor at <4 years for patients with GEJC, and <5 years for patients with GC. Median rwOS for patients who did not have surgery planned was similar to the reported OS in the GARIBALDI study (15.1 months); however, for patients with GC/GEJC who had surgery planned (including those who had surgery cancelled due to progression), median rwOS was longer than that reported in the GARIBALDI study (24.1 months) [18]. This study included data up to October 2023 versus data up to August 2022 in the GARIBALDI study, which could suggest improving outcomes for patients with GC/GEJC who undergo surgery.

A limitation of this study is that the defined endpoints of rwEFS and rwOS do not align completely with those typically used in clinical trials, which include structured visit and scan timings. This introduces the potential for incomplete or delayed event capture and could impact the accuracy of survival estimates. Furthermore, an absence of specific recurrence data or cause-specific survival data may limit the interpretation of the findings by the inability to differentiate between deaths directly attributable to cancer and those resulting from other causes. Further analysis, such as a multivariate analysis adjusting for confounding factors, would help to reinforce the conclusions, as the descriptive nature of this study limits the current findings.

## 5. Conclusions

The results from this real-world study suggest that the use of recommended perioperative treatment regimens for patients with resectable GC/GEJC was lower than expected based on recommended clinical guidance at the time of the study period; however, the emergence of FLOT as a preferred treatment option since 2018 was apparent. In addition, median rwEFS and rwOS remained poor. As such, these results highlight the need to optimize perioperative treatments to improve long-term outcomes in patients with resectable GC/GEJC. In this rapidly developing field, studies of combination therapies—such as the MATTERHORN study—could have a significant impact on the treatment landscape for resectable GC/GEJC in the near future, leading to a shift towards the use of immunotherapies in the perioperative setting.

## Figures and Tables

**Table 1:** Patient demographics and clinical characteristics for patients with GC or GEJC.

	GC (N = 901)	GEJC (N = 816)
Year of index		
2016	134 (14.9%)	124 (15.2%)
2017	169 (18.8%)	117 (14.3%)
2018	159 (17.6%)	144 (17.6%)
2019	128 (14.2%)	125 (15.3%)
2020	117 (13.0%)	103 (12.6%)
2021	104 (11.5%)	109 (13.4%)
2022	89 (9.9%)	94 (11.5%)
2023	NR	NR
Median age, years (interquartile range)	68.0 (59.0–76.0)	69.0 (61.0–75.0)
Age group		
<50 years	91 (10.1%)	46 (5.6%)
50–69 years	385 (42.7%)	389 (47.7%)
70–75 years	179 (19.9%)	192 (23.5%)
≥76 years	246 (27.3%)	189 (23.2%)
Sex		
Male	562 (62.4%)	680 (83.3%)
Female	339 (37.6%)	136 (16.7%)
Ethnicity		
White	277 (30.7%)	516 (63.2%)
Hispanic or Latino	151 (16.8%)	27 (3.3%)
Black or African American	103 (11.4%)	16 (2.0%)
Asian	74 (8.2%)	9 (1.1%)
Other race	44 (4.9%)	35 (4.3%)
Unknown race	29 (3.2%)	25 (3.1%)
Unknown	223 (24.8%)	188 (23.0%)
BMI group		
Underweight	62 (6.9%)	28 (3.4%)
Normal	359 (39.8%)	244 (29.9%)
Overweight	282 (31.3%)	270 (33.1%)
Obese	191 (21.2%)	272 (33.3%)
Unknown	7 (0.8%)	NR
Practice type		
Academic	116 (12.9%)	164 (20.1%)
Community	785 (87.1%)	652 (79.9%)
Tumor stage *		
T1	43 (4.8%)	23 (2.8%)
T2	187 (20.8%)	208 (25.5%)
T3	458 (50.8%)	555 (68.0%)
T4	213 (23.6%)	30 (3.7%)
ECOG PS		
0	299 (33.2%)	287 (35.2%)
1	202 (22.4%)	220 (27.0%)
2	56 (6.2%)	50 (6.1%)
3	9 (1.0%)	7 (0.9%)
4	NR	NR
Unknown	331 (36.7%)	252 (30.9%)
Histology		
Adenocarcinoma	877 (97.3%)	789 (96.7%)
Adenosquamous	NR	NR
Other	19 (2.1%)	6 (0.7%)
Squamous cell carcinoma	NR	20 (2.5%)
Unknown/not documented	NR	NR
Surgical resection		
Yes	641 (71.1%)	391 (47.9%)
No	260 (28.9%)	425 (52.1%)
Type of resection		
Esophagectomy	NR	105 (26.9%)
Esophagogastrectomy	27 (4.2%)	268 (68.5%)
Gastrectomy NOS	13 (2.0%)	NR
Other	10 (1.6%)	18 (4.6%)
Subtotal (partial) gastrectomy	419 (65.4%)	NR
Total gastrectomy	170 (26.5%)	NR
Unknown/not documented	NR	NR

Instances are listed as NR where the number of patients was ≤5 (to protect patient confidentiality) or where the number of patients was 0. * Includes 33 patients (GC = 28 patients; GEJC = 5 patients) with T4b tumor stage. Abbreviations: BMI, body mass index; ECOG PS, Eastern Cooperative Oncology Group performance status; GC, gastric cancer; GEJC, gastroesophageal junction cancer; NOS, not otherwise specified; NR, not reported.

**Table 2:** Grouped treatment patterns * for patients with GC.

	Neoadjuvant Treatment Only (N = 100)	Adjuvant Treatment Only (N = 169)	Neoadjuvant and Adjuvant Treatments (N = 160)	
			Neoadjuvant	Adjuvant
FLOT	43 (43.0%)	NR	86 (53.8%)	69 (43.1%)
Chemoradiotherapy	10 (10.0%)	67 (39.6%)	NR	14 (8.8%)
FOLFOX	19 (19.0%)	28 (16.6%)	31 (19.4%)	31 (19.4%)
Doublet chemotherapy	19 (19.0%)	34 (20.1%)	20 (12.5%)	26 (16.3%)
Chemotherapy plus HER2-targeted therapy	NR	NR	9 (5.6%)	6 (3.8%)
Other chemotherapy	7 (7.0%)	26 (15.4%)	9 (5.6%)	11 (6.9%)
Radiotherapy	NR	7 (4.1%)	NR	NR

Instances are listed as NR where the number of patients was ≤5 (to protect patient confidentiality) or where the number of patients was 0. * Data for the following treatment patterns are not presented as there were ≤5 patients in all treatment groups: nivolumab; chemoradiotherapy plus HER2-targeted therapy; chemotherapy plus immunotherapy; HER2-targeted therapy; and other immunotherapy. Abbreviations: FLOT, 5-fluorouracil, leucovorin, oxaliplatin, and docetaxel; FOLFOX, folinic acid (leucovorin), 5-fluorouracil, and oxaliplatin; GC, gastric cancer; HER2, human epidermal growth factor receptor 2; NR, not reported.

**Table 3:** Grouped treatment patterns * for patients with GEJC.

	Neoadjuvant Treatment Only (N = 274)	Adjuvant Treatment Only (N = 22)	Neoadjuvant and Adjuvant Treatments (N = 68)	
			Neoadjuvant	Adjuvant
FLOT	13 (4.7%)	NR	9 (13.2%)	7 (10.3%)
Chemoradiotherapy	182 (66.4%)	12 (54.5%)	46 (67.6%)	NR
FOLFOX	NR	NR	NR	13 (19.1%)
Doublet chemotherapy	37 (13.5%)	NR	8 (11.8%)	7 (10.3%)
Nivolumab	NR	NR	NR	31 (45.6%)
Other chemotherapy	6 (2.2%)	NR	NR	NR
Radiotherapy	31 (11.3%)	NR	NR	NR

Instances are listed as NR where the number of patients was ≤5 (to protect patient confidentiality) or where the number of patients was 0. * Data for the following treatment patterns are not presented as there were ≤5 patients in all treatment groups: chemotherapy plus HER2-targeted therapy; chemoradiotherapy plus HER2-targeted therapy; chemotherapy plus immunotherapy; HER2-targeted therapy; and other immunotherapy. Abbreviations: FLOT, fluorouracil, leucovorin, oxaliplatin, and docetaxel; FOLFOX, folinic acid (leucovorin), 5-fluorouracil, and oxaliplatin; GEJC, gastroesophageal junction cancer; HER2, human epidermal growth factor receptor 2; NR, not reported.

## Data Availability

The data that support the findings of this study were originated by and are the property of Flatiron Health, Inc. Requests for data sharing by license or by permission for the specific purpose of replicating results in this manuscript can be submitted to publicationsdataaccess@flatiron.com.

## Supplementary Materials

The following supporting information can be downloaded at: https://www.mdpi.com/article/10.3390/cancers17213546/s1. Figure S1. Kaplan–Meier curves of rwEFS for patients with GC or GEJC who underwent surgery or had surgery cancelled and had (a) ECOG PS of 0, (b) ECOG PS of 1, and (c) ECOG PS of ≥2. Figure S2. Kaplan–Meier curves of rwEFS for patients with GC or GEJC who underwent surgery or had surgery cancelled and had (a) stage T2 disease, (b) stage T3 disease, or (c) stage T4 disease. Figure S3. Kaplan–Meier curves of OS for patients with GC or GEJC who underwent surgery or had surgery cancelled and had (a) ECOG PS of 0, (b) ECOG PS of 1, and (c) ECOG PS of ≥2. Figure S4. Kaplan–Meier curves of OS for patients with GC or GEJC who underwent surgery or had surgery cancelled and had (a) stage T2 disease, (b) stage T3 disease, or (c) stage T4 disease. Table S1. Cohort attrition table. Table S2. Demographics and clinical characteristics for patients with GC by treatment setting. Table S3. Patient demographics and clinical characteristics for patients with GEJC by treatment setting. Table S4. Neoadjuvant therapy regimens received in patients diagnosed after 2018. Table S5. Adjuvant therapy regimens received in patients diagnosed after 2018.

## Abbreviations

The following abbreviations are used in this manuscript:

BMI	body mass index
CI	confidence interval
CROSS	41.4 Gy plus carboplatin/paclitaxel
CTLA-4	cytotoxic T-lymphocyte-associated antigen 4
dMMR	mismatch repair deficient
ECF	epirubicin, cisplatin, and infused 5-fluorouracil
ECOG PS	Eastern Cooperative Oncology Group performance status
ECX	capecitabine plus cisplatin and epirubicin
FLOT	5-fluorouracil, leucovorin, oxaliplatin, and docetaxel
FOLFOX	folinic acid (leucovorin), 5-fluorouracil, and oxaliplatin
GC	gastric cancer
GEJC	gastroesophageal junction cancer
HER2	human epidermal growth factor receptor 2
HR	hazard ratio
IQR	interquartile range
MSI-H	microsatellite instability-high
NCCN	National Comprehensive Cancer Network^®^
NE	not evaluable
NOS	not otherwise specified
NR	not reported
OS	overall survival
PD-1	programmed cell death-1
rwEFS	real-world event-free survival
rwOS	real-world overall survival
TNM	Tumor, Node, and Metastasis
US	United States
