# Supplementary material for: Real-World Treatment Patterns and Outcomes Amongst Patients with Resectable Gastric and Gastroesophageal Junction Cancer in the United States
Source: Cancers (Basel). 2025 Nov 1;17(21):3546. doi: 10.3390/cancers17213546 (PMC12609215; doi:10.3390/cancers17213546)
Supplement: Supplementary file 1 [file cancers-17-03546-s001.zip › cancers-3849330-supplementary.pdf]

Supplementary Materials

Figure S1. Kaplan–Meier curves of rwEFS for patients with GC or GEJC who underwent surgery or had surgery cancelled and had (a) ECOG PS of 0, (b) ECOG PS of 1, and (c) ECOG PS of ≥2.

(a)

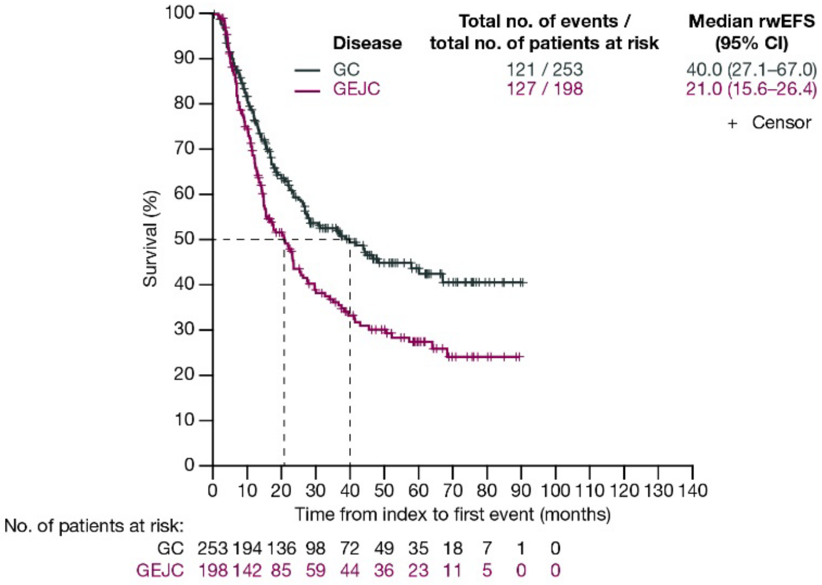

Disease
Total no. of events / total no. of patients at risk
Median rwEFS (95% CI)
GC 121 / 253 40.0 (27.1–67.0)
GEJC 127 / 198 21.0 (15.6–26.4)
+ Censor
Survival (%)
0 10 20 30 40 50 60 70 80 90 100
0 10 20 30 40 50 60 70 80 90 100 110 120 130 140
Time from index to first event (months)
No. of patients at risk:
GC 253 194 136 98 72 49 35 18 7 1 0
GEJC 198 142 85 59 44 36 23 11 5 0 0

(b)

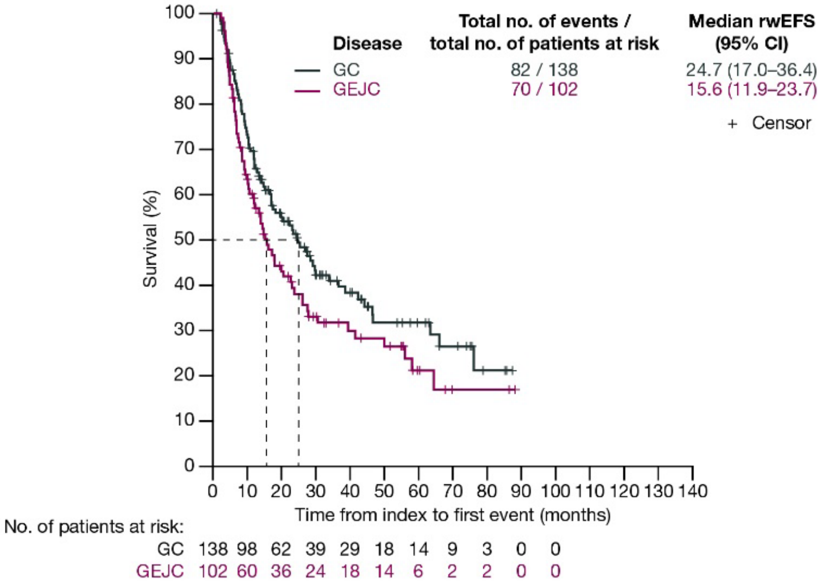

Disease
Total no. of events / total no. of patients at risk
Median rwEFS (95% CI)
GC 82 / 138 24.7 (17.0–36.4)
GEJC 70 / 102 15.6 (11.9–23.7)
+ Censor
Survival (%)
0 10 20 30 40 50 60 70 80 90 100
0 10 20 30 40 50 60 70 80 90 100 110 120 130 140
Time from index to first event (months)
No. of patients at risk:
GC 138 98 62 39 29 18 14 9 3 0 0
GEJC 102 60 36 24 18 14 6 2 2 0 0

(c)

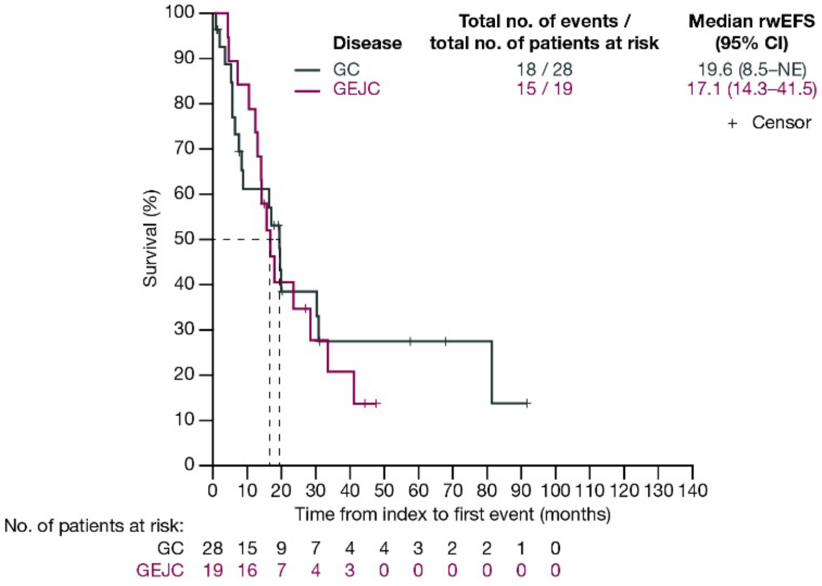

Disease
Total no. of events / total no. of patients at risk
Median rwEFS (95% CI)
GC 18 / 28 19.6 (8.5–NE)
GEJC 15 / 19 17.1 (14.3–41.5)
+ Censor
Survival (%)
0 10 20 30 40 50 60 70 80 90 100
0 10 20 30 40 50 60 70 80 90 100 110 120 130 140
Time from index to first event (months)
No. of patients at risk:
GC 28 15 9 7 4 4 3 2 2 1 0
GEJC 19 16 7 4 3 0 0 0 0 0 0

An event was defined as any documented disease progression, locoregional or distant disease recurrence, or death.

*Included patients who had surgery cancelled due to disease progression.

ECOG, Eastern Cooperative Oncology Group; NE, not evaluable.

Figure S2. Kaplan–Meier curves of rwEFS for patients with GC or GEJC who underwent surgery or had surgery cancelled and had (a) stage T2 disease, (b) stage T3 disease, or (c) stage T4 disease.

(a)

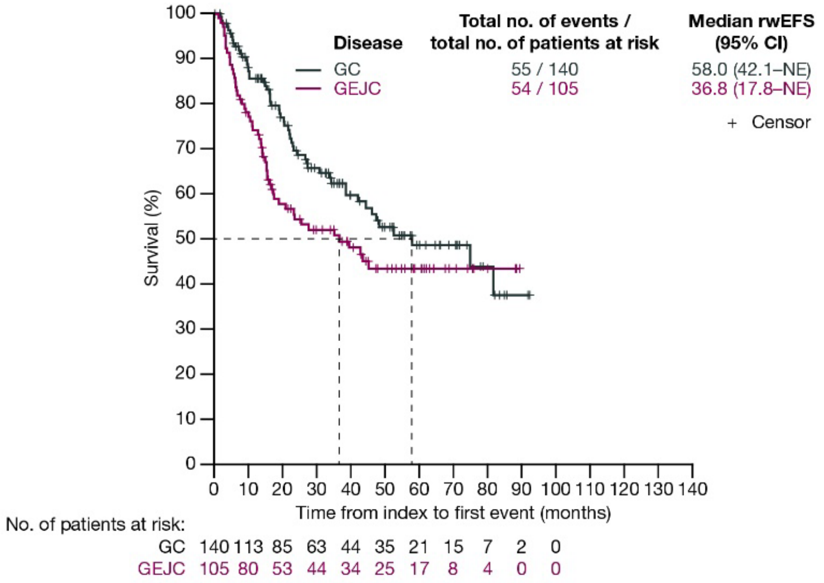

Disease
Total no. of events / total no. of patients at risk
Median rwEFS (95% CI)
GC 55 / 140 58.0 (42.1–NE)
GEJC 54 / 105 36.8 (17.8–NE)
+ Censor
Survival (%)
Time from index to first event (months)
No. of patients at risk:
GC 140 113 85 63 44 35 21 15 7 2 0
GEJC 105 80 53 44 34 25 17 8 4 0 0

(b)

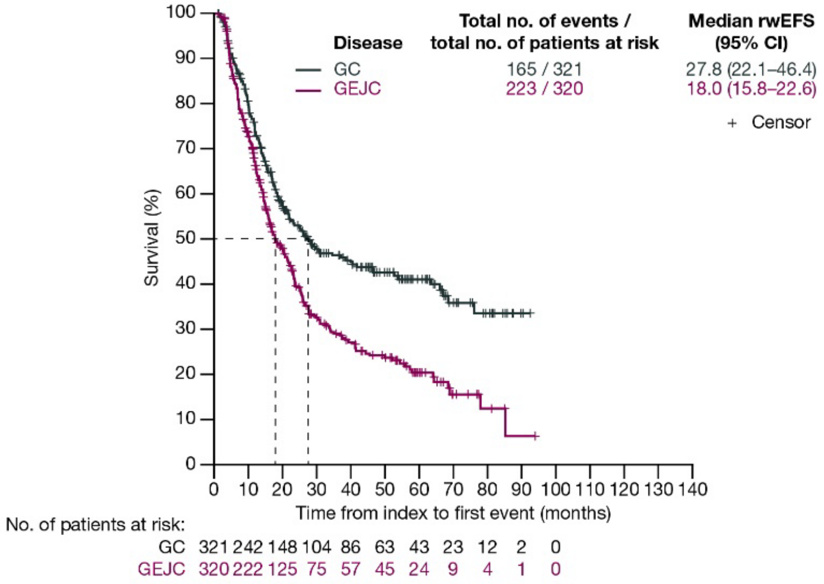

Disease
Total no. of events / total no. of patients at risk
Median rwEFS (95% CI)
GC 165 / 321 27.8 (22.1–46.4)
GEJC 223 / 320 18.0 (15.8–22.6)
+ Censor
Survival (%)
Time from index to first event (months)
No. of patients at risk:
GC 321 242 148 104 86 63 43 23 12 2 0
GEJC 320 222 125 75 57 45 24 9 4 1 0

(c)

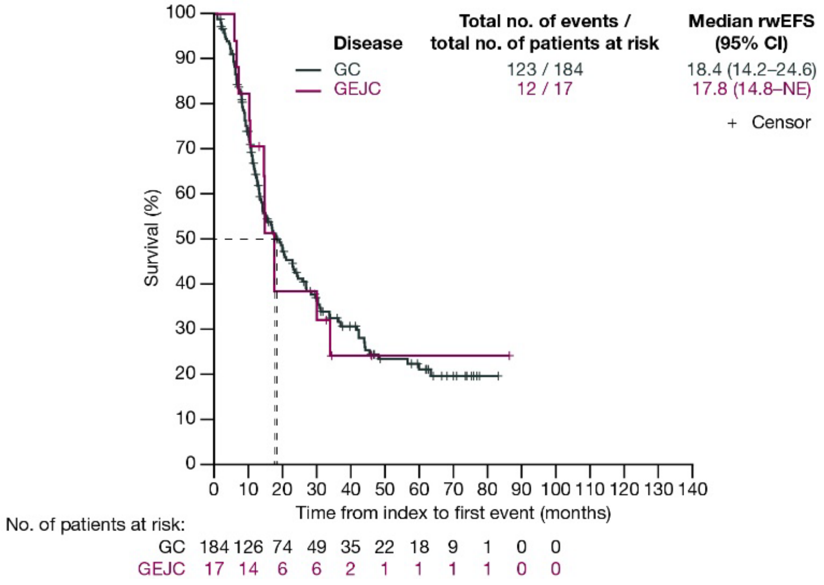

Disease
Total no. of events / total no. of patients at risk
Median rwEFS (95% CI)
GC 123 / 184 18.4 (14.2–24.6)
GEJC 12 / 17 17.8 (14.8–NE)
+ Censor
Survival (%)
Time from index to first event (months)
No. of patients at risk:
GC 184 126 74 49 35 22 18 9 1 0 0
GEJC 17 14 6 6 2 1 1 1 1 0 0

An event was defined as any documented disease progression, locoregional or distant disease recurrence, or death.

*Included patients who had surgery cancelled due to disease progression.

NE, not evaluable.

Figure S3. Kaplan–Meier curves of OS for patients with GC or GEJC who underwent surgery or had surgery cancelled and had (a) ECOG PS of 0, (b) ECOG PS of 1, and (c) ECOG PS of ≥2.

(a)

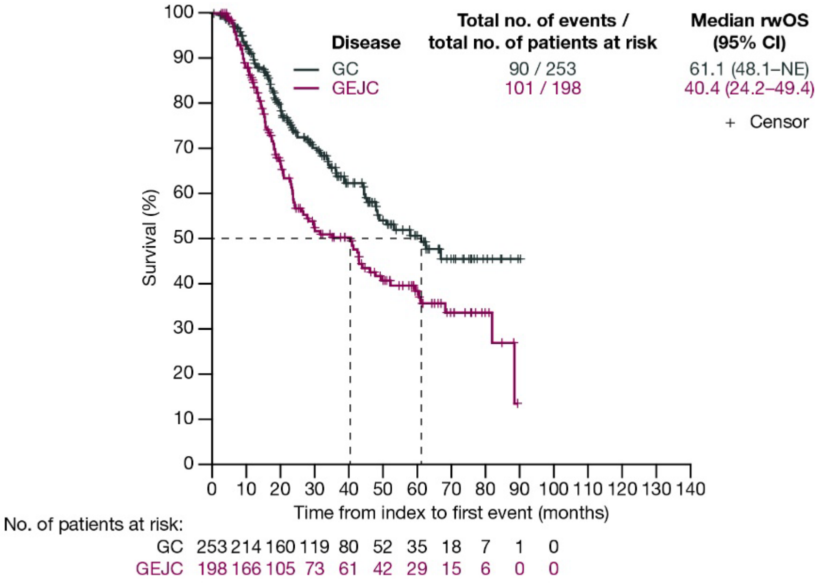

Disease
Total no. of events / total no. of patients at risk
Median rwOS (95% CI)
GC 90 / 253 61.1 (48.1–NE)
GEJC 101 / 198 40.4 (24.2–49.4)
+ Censor
Survival (%)
Time from index to first event (months)
No. of patients at risk:
GC 253 214 160 119 80 52 35 18 7 1 0
GEJC 198 166 105 73 61 42 29 15 6 0 0

(b)

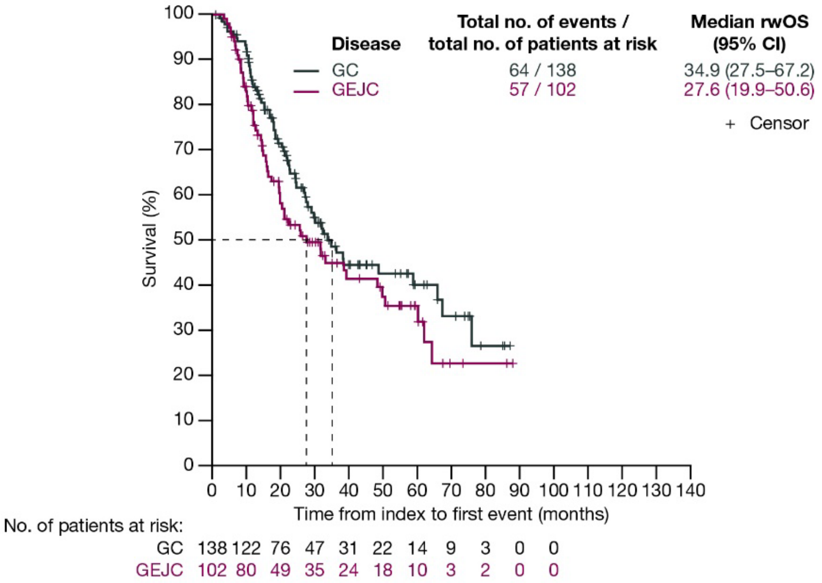

Disease
Total no. of events / total no. of patients at risk
Median rwOS (95% CI)
GC 64 / 138 34.9 (27.5–67.2)
GEJC 57 / 102 27.6 (19.9–50.6)
+ Censor
Survival (%)
Time from index to first event (months)
No. of patients at risk:
GC 138 122 76 47 31 22 14 9 3 0 0
GEJC 102 80 49 35 24 18 10 3 2 0 0

(c)

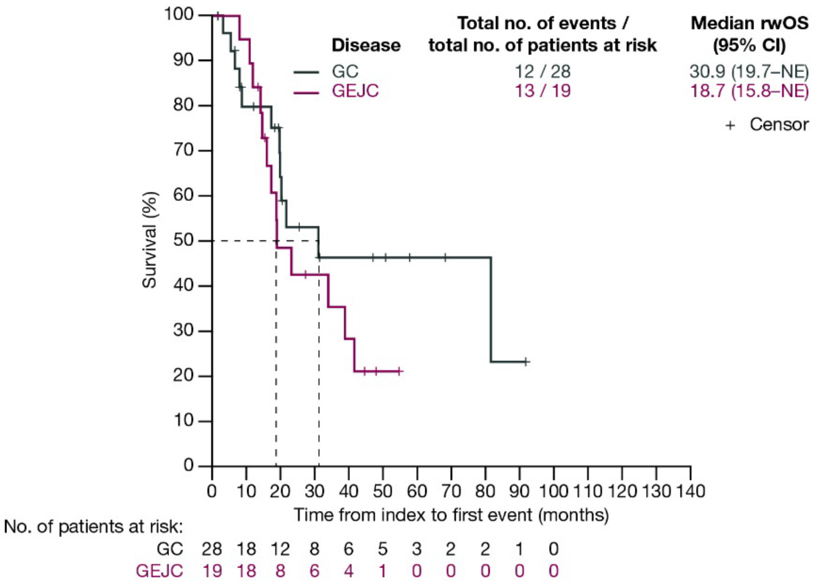

Disease
Total no. of events / total no. of patients at risk
Median rwOS (95% CI)
GC 12 / 28 30.9 (19.7–NE)
GEJC 13 / 19 18.7 (15.8–NE)
+ Censor
Survival (%)
Time from index to first event (months)
No. of patients at risk:
GC 28 18 12 8 6 5 3 2 2 1 0
GEJC 19 18 8 6 4 1 0 0 0 0 0

*Included patients who had surgery cancelled due to disease progression.

ECOG, Eastern Cooperative Oncology Group; NE, not evaluable.

Figure S4. Kaplan–Meier curves of OS for patients with GC or GEJC who underwent surgery or had surgery cancelled and had (a) stage T2 disease, (b) stage T3 disease, or (c) stage T4 disease.

(a)

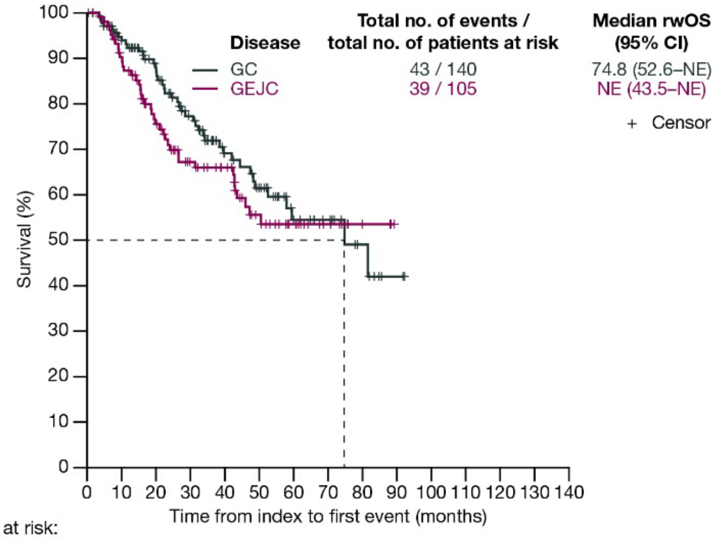

No. of patients at risk:

	0	10	20	30	40	50	60	70	80	90	100
GC	140	120	97	73	47	37	20	15	7	2	0
GEJC	105	91	68	52	42	27	18	9	3	0	0

(b)

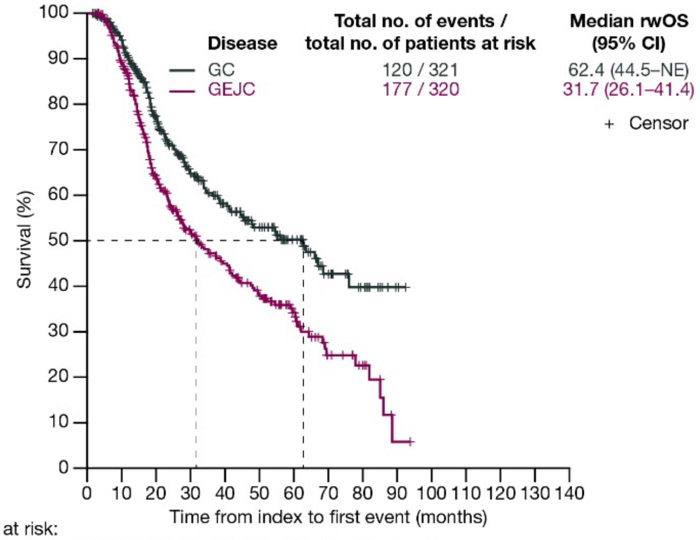

No. of patients at risk:

	0	10	20	30	40	50	60	70	80	90	100
GC	321	277	188	130	98	68	45	22	12	2	0
GEJC	320	269	163	112	86	63	36	16	9	1	0

(c)

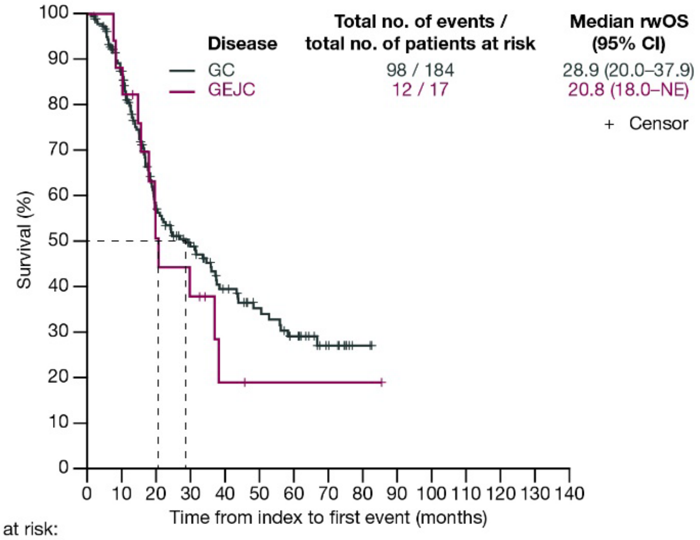

No. of patients at risk:

	0	10	20	30	40	50	60	70	80	90	100
GC	184	145	82	59	40	29	21	10	2	0	0
GEJC	17	15	9	7	2	1	1	1	1	0	0

*Included patients who had surgery cancelled due to disease progression.

NE, not evaluable.

Table S1. Cohort attrition table.

	Full data set, n	Patients with GC, n	Patients with GEJC, n
Starting population	18,150	5970	4011
Diagnosis with GC or GEJC	9981	5970	4011
Index diagnosis between January 01, 2016 and January 01, 2023	5648	3398	2250
Have TNM stage information	2481	1316	1165
TNM stage (T2 or higher, N0–3, and M0) or (T0–4, N1–3, and M0)	2037	1056	981
Age >18 years at diagnosis	2037	1056	981
Not clinical study participants	1972	1029	943
No cancer or metastatic disease diagnosis prior to index diagnosis	1734	910	824
Not diagnosed with gastrointestinal stromal tumors	1725	904	821
Not diagnosed with lymphoma	1717	901	816
No missing sex information	1717	901	816

All data points are based on Flatiron Health Enhanced Datamart (Flatiron Health®, New York, NY, USA), where the end of data window was October 31, 2023.

Abbreviations: GC, gastric cancer; GEJC, gastroesophageal junction cancer; n, number of patients; TNM, Tumor, Node, and Metastasis.

Table S2. Demographics and clinical characteristics for patients with GC by treatment setting.

	**Neoadjuvant treatment only (N = 100)**	**Adjuvant treatment only (N = 169)**	**Neoadjuvant and adjuvant treatment (N = 160)**
Year of index			
2016	15 (15.0%)	37 (21.9%)	18 (11.3%)
2017	14 (14.0%)	35 (20.7%)	29 (18.1%)
2018	15 (15.0%)	26 (15.4%)	36 (22.5%)
2019	11 (11.0%)	23 (13.6%)	17 (10.6%)
2020	22 (22.0%)	19 (11.2%)	20 (12.5%)
2021	11 (11.0%)	16 (9.5%)	27 (16.9%)
2022	12 (12.0%)	12 (7.1%)	13 (8.1%)
2023	NR	NR	NR
Median age, years (interquartile range)	66.0 (56.5–74.0)	67.0 (58.0–74.0)	64.0 (57.0–72.0)
Age group			
<50 years	11 (11.0%)	17 (10.1%)	19 (11.9%)
50–69 years	50 (50.0%)	83 (49.1%)	86 (53.8%)
70–75 years	17 (17.0%)	36 (21.3%)	34 (21.3%)
≥76 years	22 (22.0%)	33 (19.5%)	21 (13.1%)
Sex			
Male	70 (70.0%)	104 (61.5%)	104 (65.0%)
Female	30 (30.0%)	65 (38.5%)	56 (35.0%)
Ethnicity			
White	36 (36.0%)	38 (22.5%)	48 (30.0%)
Hispanic or Latino	12 (12.0%)	31 (18.3%)	19 (11.9%)
Black or African American	11 (11.0%)	20 (11.8%)	18 (11.3%)
Asian	8 (8.0%)	20 (11.8%)	20 (12.5%)
Other race	6 (6.0%)	8 (4.7%)	6 (3.8%)
Unknown race	NR	NR	8 (5.0%)
**Missing**	24 (24.0%)	47 (27.8%)	41 (25.6%)
BMI group			
Underweight	NR	12 (7.1%)	NR
Normal	36 (36.0%)	75 (44.4%)	54 (33.8%)
Overweight	35 (35.0%)	49 (29.0%)	56 (35.0%)
Obese	24 (24.0%)	33 (19.5%)	45 (28.1%)
Unknown	NR	NR	NR
Practice type			
Academic	11 (11.0%)	11 (6.5%)	24 (15.0%)
Community	89 (89.0%)	158 (93.5%)	136 (85.0%)
Tumor stage			
T1	NR	23 (13.6%)	NR
T2	17 (17.0%)	22 (13.0%)	48 (30.0%)
T3	72 (72.0%)	62 (36.7%)	87 (54.4%)
T4	9 (9.0%)	62 (36.7%)	24 (15.0%)
Histology*			
Adenocarcinoma	98 (98.0%)	165 (97.6%)	150 (93.8%)
Other	NR	NR	8 (5.0%)
Surgical resection			
Yes	100 (100.0%)	169 (100.0%)	160 (100.0%)

Type of resection			
Esophagogastrectomy	14 (14.0%)	NR	9 (5.6%)
Gastrectomy NOS	NR	NR	NR
Other	NR	NR	NR
Subtotal (partial) gastrectomy	42 (42.0%)	128 (75.7%)	100 (62.5%)
Total gastrectomy	37 (37.0%)	33 (19.5%)	47 (29.4%)
Unknown/not documented	NR	NR	NR
ECOG PS			
0	49 (49.0%)	58 (34.3%)	85 (53.1%)
1	28 (28.0%)	30 (17.8%)	33 (20.6%)
2	NR	7 (4.1%)	NR
3	NR	NR	NR
4	NR	NR	NR
Unknown	20 (20.0%)	74 (43.8%)	41 (25.6%)

Instances are listed as NR where the number of patients was ≤5 (to protect patient confidentiality) or where the number of patients was 0.

*Data for the following histology groups are not presented as there were ≤5 patients in all groups: adenosquamous; and unknown/undocumented.

Abbreviations: BMI, body mass index; ECOG PS, Eastern Cooperative Oncology Group performance status; GC, gastric cancer; NOS, not otherwise specified; NR, not reported.

Table S3. Patient demographics and clinical characteristics for patients with GEJC by treatment setting.

	**Neoadjuvant treatment only (N = 274)**	**Adjuvant treatment only (N = 22)**	**Neoadjuvant plus adjuvant treatment (N = 68)**
Year of index			
2016	46 (16.8%)	NR	14 (20.6%)
2017	54 (19.7%)	NR	NR
2018	60 (21.9%)	NR	12 (17.6%)
2019	38 (13.9%)	NR	NR
2020	29 (10.6%)	NR	10 (14.7%)
2021	22 (8.0%)	6 (27.3%)	15 (22.1%)
2022	25 (9.1%)	NR	12 (17.6%)
Median age, years (interquartile range)	65.5 (59.0–71.0)	69.0 (66.0–72.0)	65.5 (58.0–70.0)
Age group			
<50 years	15 (5.5%)	NR	7 (10.3%)
50–69 years	170 (62.0%)	11 (50.0%)	42 (61.8%)
70–75 years	65 (23.7%)	11 (50.0%)	13 (19.1%)
≥76 years	24 (8.8%)	NR	6 (8.8%)
Sex			
Male	242 (88.3%)	17 (77.3%)	62 (91.2%)
Female	32 (11.7%)	NR	6 (8.8%)
Ethnicity			
White	187 (68.2%)	18 (81.8%)	43 (63.2%)
Hispanic or Latino	10 (3.6%)	NR	NR
Black or African American	NR	NR	NR
Asian	NR	NR	NR
Other race	11 (4.0%)	NR	NR
Unknown race	NR	NR	NR
Unknown	55 (20.1%)	NR	14 (20.6%)
BMI group			
Underweight	7 (2.6%)	NR	NR
Normal	71 (25.9%)	8 (36.4%)	15 (22.1%)
Overweight	94 (34.3%)	7 (31.8%)	24 (35.3%)
Obese	102 (37.2%)	7 (31.8%)	27 (39.7%)
Unknown	NR	NR	NR
Practice type			
Academic	54 (19.7%)	NR	NR
Community	220 (80.3%)	20 (90.9%)	64 (94.1%)
Tumor stage			
T1	6 (2.2%)	NR	NR
T2	56 (20.4%)	9 (40.9%)	18 (26.5%)
T3	202 (73.7%)	9 (40.9%)	45 (66.2%)
T4	10 (3.6%)	NR	NR
Histology*			
Adenocarcinoma	268 (97.8%)	22 (100.0%)	66 (97.1%)
Surgical resection			
Yes	274 (100.0%)	22 (100.0%)	68 (100.0%)
Type of resection			
Esophagectomy	79 (28.8%)	NR	18 (26.5%)

Esophagogastrectomy	182 (66.4%)	17 (77.3%)	48 (70.6%)
Other	13 (4.7%)	NR	NR
ECOG PS, n (%)			
0	130 (47.4%)	6 (27.3%)	37 (54.4%)
1	59 (21.5%)	NR	18 (26.5%)
2	NR	NR	NR
3	NR	NR	NR
Unknown	77 (28.1%)	12 (54.5%)	8 (11.8%)

Instances are listed as NR where the number of patients was ≤5 (to protect patient confidentiality) or where the number of patients was 0.

*Data for the following histology groups are not presented as there were ≤5 patients in all groups: adenosquamous; squamous cell carcinoma; and other.

Abbreviations: BMI, body mass index; ECOG PS, Eastern Cooperative Oncology Group performance status; GEJC, gastroesophageal junction cancer; NR, not reported.

Table S4. Neoadjuvant therapy regimens received in patients diagnosed after 2018

	GC (N=598)	GEJC (N=575)
Available data	n=408	n=258
No treatment	224 (54.9%)	31 (12.0%)
CRT	NR	143 (55.4%)
FLOT	118 (28.9%)	22 (8.5%)
FOLFOX	34 (8.3%)	6 (2.3%)
Doublet chemotherapy	10 (2.5%)	27 (10.5%)
Radiotherapy	NR	23 (8.9%)
Other Chemotherapy	8 (2.0%)	NR
Chemotherapy + HER2	6 (1.5%)	NR

Instances are listed as NR where the number of patients was ≤5 (to protect patient confidentiality) or where the number of patients was 0.

*Data for the following treatment patterns are not presented as there were ≤5 patients in all treatment groups: chemoradiotherapy plus HER2-targeted therapy; chemotherapy plus immunotherapy. Data were missing for 190 GC patients and 317 GEJC patients.

Abbreviations: FLOT, 5-fluorouracil, leucovorin, oxaliplatin, and docetaxel; FOLFOX, folinic acid (leucovorin), 5-fluorouracil, and oxaliplatin; GC, gastric cancer; GEJC, gastroesophageal junction cancer; HER2, human epidermal growth factor receptor 2; NR, not reported.

Table S5. Adjuvant therapy regimens received in patients diagnosed after 2018

	GC (N=598)	GEJC (N=575)
Available data	n=408	n=258
No treatment	198 (48.5%)	191 (74.0%)
FLOT	60 (14.7%)	7 (2.7%)
FOLFOX	44 (10.8%)	13 (5.0%)
CRT	35 (8.6%)	7 (2.7%)
Nivolumab	NR	32 (12.4%)
Doublet chemotherapy	32 (7.8%)	NR
Other Chemotherapy	28 (6.9%)	NR

Instances are listed as NR where the number of patients was ≤5 (to protect patient confidentiality) or where the number of patients was 0.

*Data for the following treatment patterns are not presented as there were ≤5 patients in all treatment groups: chemotherapy plus HER2-targeted therapy; radiotherapy; chemotherapy plus immunotherapy; HER2-targeted therapy; and other immunotherapy. Data were missing for 190 GC patients and 317 GEJC patients.

Abbreviations: CRT, chemoradiotherapy; FLOT, 5-fluorouracil, leucovorin, oxaliplatin, and docetaxel; FOLFOX, folinic acid (leucovorin), 5-fluorouracil, and oxaliplatin; GC, gastric cancer; GEJC, gastroesophageal junction cancer; HER2, human epidermal growth factor receptor 2; NR, not reported.
